# The impact of COVID-19 on knowledge, attitude, and infection control behaviors among dentists

**DOI:** 10.1186/s12903-021-01946-w

**Published:** 2021-11-19

**Authors:** Hsin-Chung Cheng, Yan-Ju Chang, Shin-Ru Liao, Pallop Siewchaisakul, Sam Li-Sheng Chen

**Affiliations:** 1 School of Dentistry, College of Oral Medicine, Taipei Medical University, Taipei, Taiwan; 2 Division of Orthodontics, Department of Dentistry, Taipei Medical University Hospital, Taipei, Taiwan; 3 Department of Dental Education Development, Graduate School of Medical and Dental Sciences, Tokyo Medical and Dental University, Tokyo, Japan; 4 Faculty of Public Health, Chiang Mai University, Chiang Mai, Thailand; 5 School of Oral Hygiene, College of Oral Medicine, Taipei Medical University, No.250, Wuxing St., Xinyi Dist, Taipei, 110 Taiwan

**Keywords:** COVID-19, Dentistry, Infection control, Taiwan

## Abstract

**Background:**

This study seeks to elucidate the impact of COVID-19 on knowledge, attitude, and infection control behaviors among dentists.

**Methods:**

Changes in knowledge, attitude, and infection control behaviors reported in 2020 (COVID-19 period) were compared to the historical control of the non-COVID-19 period in 2018. A proportional random sampling method was used to select the study samples from 400 dental institutions. The response rate was 69% in 2018 and 62.8% in 2020. A total of 276 dentists in 2018 and 251 dentists in 2020 responded to this questionnaire. Multiple logistic regression was used to assess the associations between factors and recommended infection control practices.

**Results:**

High rates of correct COVID-19 knowledge (94.76%), fears of being infected with the virus (94%) and use of personal protective equipment (mask, glove and protection gown; 95%) were reported. We found that knowledge regarding environmental infection control, HIV transmission, and the window of HIV transmission were significantly higher in the post-COVID-19 period compared with the pre-COVID-19 period. High compliance rates of wearing mask, gloves and protection were reported. The number of dentists wearing a hair cap and a protective eye mask/face shield during the pandemic significantly increased compared with that noted before the COVID-19 pandemic. Factors associated with the use of a hair cap and an eye mask/face shield differed between the pre- and post-COVID-19 periods. The factors associated with compliance regarding environment infection control also differed between the pre- and post-COVID-19 periods.

**Conclusion:**

The significant impact of COVID-19 on the knowledge, attitude, and infection control behaviors among dental care workers was observed in the current study. In particular, the use of hair caps and protective eye mask or face shields as well as environmental disinfection protocols has significantly improved.

*Trial registration* TMU-JIRB: N201804006.

**Supplementary Information:**

The online version contains supplementary material available at 10.1186/s12903-021-01946-w.

## Background

The coronavirus disease 2019 (COVID-19) outbreak was initially reported in Wuhan, China. COVID-19 is an infectious disease caused by the severe acute respiratory syndrome coronavirus 2 (SARS-CoV-2) [1]. The virus quickly spread throughout the world, including Taiwan. As of September 2021, greater than 224.5 million confirmed cases of COVID-19 and over 4.6 million deaths worldwide have been reported [2] with 16,093 confirmed cases and 839 deaths in Taiwan. Notably, the COVID-19 outbreak was quite modest in Taiwan compared with other countries, and the highest daily case number was 27 in 2020 [3].

Many countries have adopted non-pharmaceutical and pharmaceutical approaches to control the COVID-19, including quarantine, physical distancing, hygiene measures and vaccine, [4]. However, new variants are emerging due to the delayed arrival of a vaccine and vaccine hesitancy [5–7]. As the result, the virus is still causing global turmoil and has not yet been effectively controlled.

The common transmission routes of COVID-19 include direct transmission (cough, sneeze, and droplet transmission) and contact transmission (contact with oral, nasal, and eye mucous membranes) [8, 9]. Inevitably, dental care workers represent a group at high risk of being infected with COVID-19. The dental care professionals’ work involves in-person communication with patients and routine dental clinical procedures. These type of work is associated with an increased opportunity for virus exposure via the described transmission routes [10, 11]. Therefore, this risk may cause fear and worry among dental staffs, especially dentists [12].

To prevent risk of the COVID-19 infection among dentists, many of disease control organization, including Taiwan Centers of Disease Control, have provided guidance for comprehensive preventive measures in the field of dentistry [11, 13, 14]. Several studies have previously reported lack of knowledge, attitudes, and perceptions of dentists regarding viral infection control [15–17], including COVID-19 [18, 19].

Taiwan experienced a SARS outbreak with approximately 680 probable cases in 2003. A study reported different of attitudes regarding common protective measures against SARS infection among nurses during and after the epidemic [20]. Furthermore, compliance with infection prevention and control is one of the important meth to minimize the risk of COVID-19 infection among healthcare, but different health professionals demonstrate different levels of compliance [21]. As previously mentioned, the highly dangerous and contagious SARS-CoV-2 virus may have an impact on changing knowledge, attitude and behavior of communicable disease control among dentists in Taiwan.

Thus, the first aim of this study is to explore and compare of the knowledge, attitude, and behavior of infection control between two periods of time, namely, before and during the COVID-19 outbreak. Second, we aim to investigate factors associated with the change in infection control compliance among dentists.

## Methods

### First surge of COVID-19 in Taiwan

**Fig. 1:**
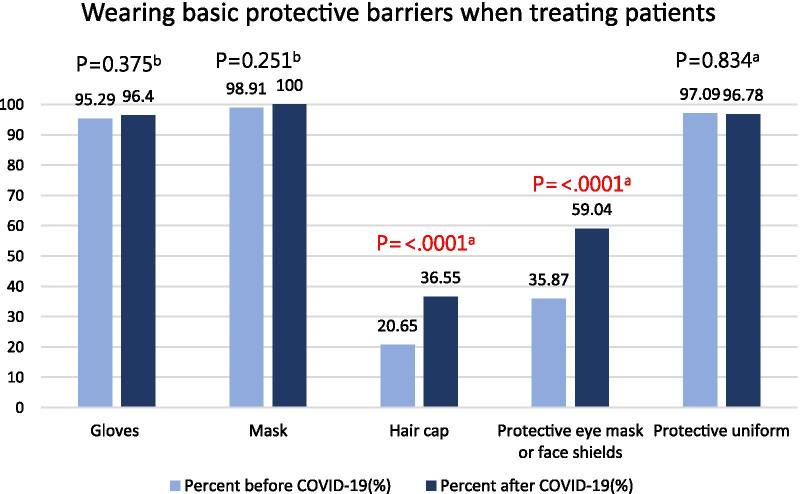
Comparison of the implementation of personal protective equipment among dentists before and after the COVID-19 outbreak. ^a^Chi-squared test. ^b^Fisher exact test

The epidemiological profile of confirmed COVID-19 cases from January to May, 2020 in Taiwan indicated the first surge with a peak on March 16th due to imported cases (see Additional file 1: Supplmental Figure 1). In total, 446 confirmed cases were noted during this period. After a series of containment measures, such as social distancing, there was a substantial decline in confirmed cases of COVID-19 until May, 2020 [22]. Due to the fast response to COVID-19, routine dental care service has been remained fully functioning and accessible.

### Study design

The differences in knowledge, attitude, and behavior of infection control among dentists before and after COVID-19 pandemic were assessed. Two investigations of knowledge, attitude, and infection control behaviors were conducted separately in 2018 and 2020. The investigation in 2018 (non-COVID-19 period) served as the control group. To obtain a nationally representative sample of dentists, a proportional random sampling method was used. The probabilities proportional to size (PPS) method was used to randomly select subjects from different, geographically distant areas of Taiwan: north, south, east, and west. The participating clinics were under no strict selection criteria, and mostly all clinics operate in a similar fashion. Based on 35% and 5% sampling fractions from hospital and clinics, respectively, a total of 400 dental institutions, including 60 dentistry hospitals and 340 dental clinics, were selected from 6965 dental institutions. We believe that our study sample was sufficiently random and representative given that clinics were chosen from six areas. An anonymous questionnaire was mailed to these dental institutions in May 2018 and April 2020, separately, and the dental institution randomly invited one dentist to complete the questionnaire in an anonymous manner. A second mailing was sent approximately one month later, and follow-up calls were made one week after the first and second mailings. We mailed questionnaires with an approximately US$3 honorarium to encourage the selected dentists to return the completed questionnaires.

### Questionnaire

The content of the questionnaire in this study was based on the questionnaire of the “Dental Infection Control Implementation and Cognitive Status Evaluation Survey” of the “2018 Commissioned Technology Research Project of the Ministry of Health and Welfare—Establishing a Self-evaluation Mechanism for Dental Infection Control Operations” in Taiwan. The questionnaire was slightly modified in 2020. All questions in the questionnaire were designed by three experts. The content of the questionnaire was reviewed and validated by the three expert reviewers. The experts conducted their review independently. Written informed consent was obtained from all study participants, and the survey was reviewed and approved by the Institutional Review Board of Taipei Medical University (TMU-JIRB: N201804006).

The questionnaire consists of four parts. The first part focuses on the characteristics of dentists and their practice settings, such as gender, age, length of dental practice experience, types of dental institutions serving, the number of dentists, dental assistants and dental chairs, and location of the medical institution. The second part assess the dentist’s awareness and attitude towards the implementation of infection control measures, including wearing personal protective equipment (PPE), such as glove, masks, hair cap, protective eye mask or face shields, and protective uniforms. The implementation of disinfection and sterilization protocols, such as sterilization of dental instruments, sterilization monitoring, surface disinfection of working tables, disinfection of impression materials, and periodic disinfection of waterlines, are also included in this section. The third section focuses on the risk of infection, the needlestick injury protocol, and information on infection control. The fourth section focuses on the dentist’s knowledge and attitude towards dental infectious diseases. To assess the respondent’s knowledge of infection control, we asked about the transmission routes of hepatitis B, C, and AIDS, proportion of hepatitis B carriers among Taiwanese adults, the relation between the rate of cirrhosis or hepatocellular carcinoma and hepatitis B infection, oral manifestations of AIDS, and the HIV screening time and window of transmission.

To assess their attitude, we asked about how the dental profession would respond if they treated a patient infected with hepatitis B or C or HIV. We also asked about their thoughts on the possibility of getting AIDS, Hepatitis B, or tuberculosis while performing dental work. We further asked about their flu vaccination status. In addition, COVID-19-related questions were added to the 2020 questionnaire.

Of the 400 questionnaires that were delivered to selected dental institutions in 2018, 276 (69%) were completed. In 2020, 400 questionnaires were delivered, and 251 dentists completed the survey with a response rate of 62.75%. No significant difference in demographic characteristics were noted between respondents of the 2018 and 2020 questionnaires according to a goodness-of-fit test (P > 0.05, data not shown). The goodness-of-fit test was conducted separately in 2018 and in 2020.

### Statistical analysis

Categorical variables were summarized as frequencies and percentages, and chi-square tests were performed to compare differences between the 2018 and 2020 surveys. Multiple logistic regression was used to assess the associations between all variables and compliance with recommended infection control practices, and the odds ratios (ORs) and 95 percent confidence intervals (CIs) were reported. A P-value of < 0.05 was considered to be statistically significant. All analyses were performed using Statistical Analysis Software (SAS, version 9.4, SAS Institute Inc., Cary, NC, USA).

## Results

### Respondent characteristics before and after the COVID-19 outbreak

As noted in Table 1, no significant difference in the background information of dentists was noted before and after the COVID-19 outbreak. The distribution of background information of dentists is similar before and after the COVID-19. Most of the participants were males over 50 years of age. Their dental practice has been operational between 21 and 30 years. Most professionals worked less than or equal to 5 days per week and treated 11–20 patients per day. Most patients were adults.

**Table 1:** Characteristics of participants before and after the COVID-19 outbreak

Characteristics of participants	Before COVID-19 (N = 276)		After COVID-19 (N = 251)		P-value
	N	%	N	%	
Gender					
Male	223	80.8	207	82.8	0.5526^a^
Female	53	19.2	43	17.2	
Age (years)					
≤ 50	96	34.99	88	35.11	0.2896^a^
51 ~ 60	82	29.91	89	35.46	
≥ 61	96	35	74	29.51	
Length of dental practice experience					
≤ 10	56	20.35	39	15.55	0.1012^a^
11 ~ 20	59	21.44	47	18.73	
21 ~ 30	95	34.56	82	32.67	
31 ~ 40	55	20	64	25.5	
> 40	10	3.62	19	7.59	
Work day per week					
≤ 5 days	172	62.76	169	67.33	0.2743^a^
> 5 days	102	37.21	82	32.67	
Number of patients treated per day					
0 ~ 10	48	17.78	54	21.6	0.3537^a^
11 ~ 20	161	59.63	150	60	
> 20	61	22.59	46	18.4	
Patient age group					
Children or teenagers (≦19 years old)	10	4.59	4	1.88	0.0637^b^
Adults (20 ~ 60 years old)	195	89.45	186	87.32	
Elderly patients (> 60 years old)	13	5.96	23	10.80	

Variables with missing values: Gender (after 1); Age (before 2); Length of practice (before 1); Working days per week (before 2); Number of patients treated per day (before 6; after 1); Patient age group (before 58; after 38)

^a^Chi-squared test

^b^Fisher exact test

### Institutional characteristics before and after the COVID-19 outbreak

As noted in Table 2, there is no significant difference in the background information of the dentist's institution before and after the COVID-19 outbreak. The institutional background information of respondents was similar before and after the COVID-19 outbreak. Most of the institutions were clinics and were not post-graduate year training institutions. Most institutions had 10 or less dentists with 0–5 dental assistants and 0–5 dental chairs and were located in Taipei.

**Table 2:** Characteristics of the participants’ institutions before and after the COVID-19 outbreak

Characteristics of the participant’s institution	Before COVID-19 (N = 276)		After COVID-19 (N = 251)		P-value^a^
	N	%	N	%	
Setting level					
Hospital	57	20.88	67	27.02	0.1004
Clinic	216	79.12	181	72.98	
PGY teaching and training institution					
Yes	76	28.68	55	22.18	0.0993
No	189	71.32	193	77.82	
Number of dentists					
1	120	43.8	115	45.82	0.6228
2 ~ 10	122	44.5	102	40.63	
> 10	32	11.62	34	13.59	
Number of dental assistants					
0 ~ 5	182	66.66	175	70.56	0.5365
6 ~ 10	49	17.96	36	14.53	
> 10	42	15.4	37	14.89	
Number of dental clinic chair					
1 ~ 5	191	69.71	184	73.3	0.1107
6 ~ 10	56	20.43	35	13.95	
> 10	27	9.75	32	12.8	
Location of medical institution					
Taipei	93	33.7	88	35.7	0.3454
Northern region	32	11.59	26	10.36	
Central Region	77	27.9	54	21.51	
Southern region	28	10.15	39	15.54	
Kaohsiung Pingtung	39	14.14	36	14.35	
Eastern Region	7	2.54	8	3.18	

Variables with missing values: Setting level (before 3; after 3); PGY teaching and training institution (before 11; after 3); Number of dentists (before 2); Number of dental chairs (before 2)

^a^Chi-squared test

### Changes in dentists’ knowledge, attitudes, and behaviors regarding infection control after the COVID-19 outbreak

#### Changes in the implementation of personal protective equipment after the COVID-19 outbreak (Fig. 1)

In terms of personal protection equipment, significant differences were noted between wearing hair caps and wearing protective eye mask or face shields (P < 0.0001), but no significant differences were noted between wearing gloves, masks, and protective uniforms. After the COVID-19 outbreak, the percentage of dentists wearing hair caps increased from 21 to 37% (p < 0.0001), and the percentage of those wearing protective eye mask and face shields increased from 36 to 59% (P < 0.0001).

#### Changes in the use of environmental protection measures by dentists after the COVID-19 outbreak (Fig. 2)

**Fig. 2:**
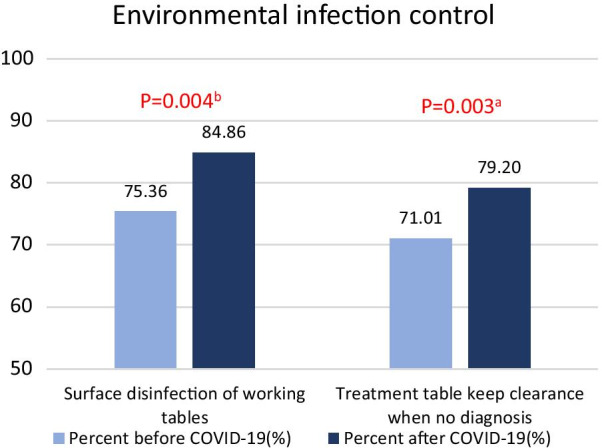
Comparison of the implementation of environmental infection control by dentists before and after the COVID-19 outbreak. ^a^Chi-squared test. ^b^Fisher exact test

In terms of environmental protection, significant differences were noted between “performing surface disinfection on the working tables after diagnosing each patient” and “keeping the treatment table clear when not diagnosing a patient”. After the COVID-19 outbreak, more dentists disinfected working tables, and the percentage increased from 75 to 85% (p = 0.004). In addition, the percentage of dentists who reported keeping the treatment table clear when not diagnosing a patient increased from 71 to 79% (P = 0.003).

#### Changes in knowledge and attitudes about dental-related infectious diseases after the COVID-19 outbreak (Fig. 3)

**Fig. 3:**
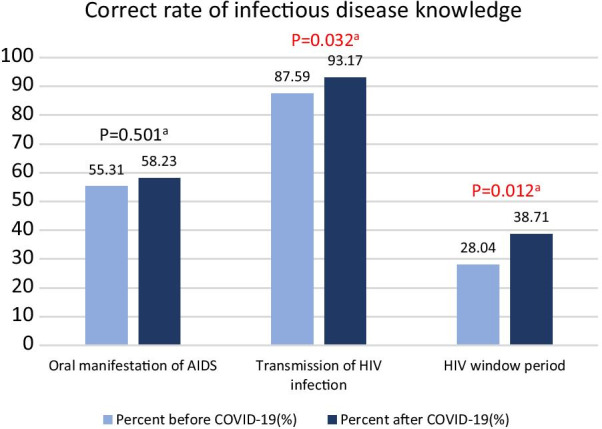
Comparison of the rate of correct infectious disease knowledge among dentists before and after the COVID-19 outbreak. ^a^Chi-squared test

Figure 3 describes the knowledge and attitudes towards dental-related infectious diseases. Significant differences in HIV-related knowledge and experience are noted, but no significant differences in other dental-related infectious diseases were observed. After the COVID-19 outbreak, the rate of correct responses regarding the transmission mode of HIV increased from 88 to 93% (P = 0.03), and that of the window of HIV transmission increased from 28 to 39% (P = 0.01).

**Fig. 4:**
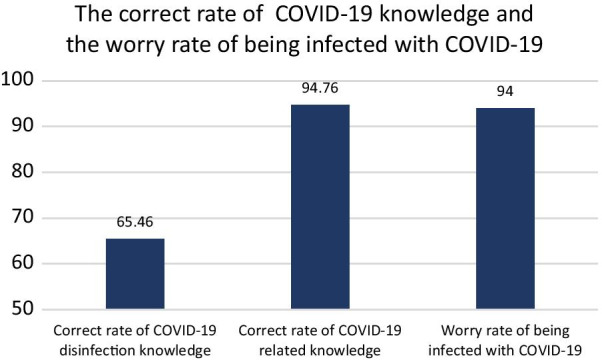
The rate of correct COVID-19 knowledge and the rate of concerns regarding COVID-19 infection among dentists

In Fig. 4, we reported the percentage of correct responses to questions regarding COVID-19 knowledge and the degree of fear associated with COVID-19 after the COVID-19 outbreak. In total, 66% and 95% of respondents provided correct responses regarding COVID-19 disinfection and other COVID-19 disinfection protocols. In total, 94% of respondents were worried about being infected with COVID-19.

### Factors affecting the application of infection control measures by dentists before and after the COVID-19 outbreak

#### Factors associated with compliance with wearing hair caps before and after the COVID-19 outbreak

Before the COVID-19 outbreak, factors, such as “getting the flu vaccine every year” and “wearing protective eye mask or face shields”, were associated with the use of hair caps by dentists (P < 0.05). Dentists who received the flu vaccine every year reported a 2.304-fold increased compliance rate of wearing hair caps compared with dentists who do not undergo regular flu vaccination (OR = 2.30; 95% CI: 1.20–4.43). Those dentists who wear a protective eye mask or face shield are also 4.395- fold (OR = 4.40; 95% CI: 2.34–8.24) more likely to wear a hair cap than those who do not wear a protective eye mask or face shields. After the COVID-19 outbreak, in addition to vaccinations and wearing protective eye mask or face shields, additional factors, including working in a hospital-level setting (OR = 5.94, 95% CI: 2.93–12.06) and using a chemical indicator (OR = 3.78, 95% CI: 1.35–10.56), were significantly associated with increased compliance of wearing a hair cap (Table 3).

**Table 3:** Factors associated with compliance with wearing hair caps before and after the COVID-19 outbreak

Variables	Before COVID-19				After COVID-19			
	Wear hair caps				Wear hair caps			
	Adjusted odds ratio		95% CI	P-value	Adjusted odds ratio		95% CI	P-value
Setting								
Hospital	–			–	1.0	–		< 0.0001
Clinic	–	–	–		5.94	2.93	12.06	
Chemical indicator (each package)								
No	–			–	1.0	–		0.0111
Yes (each package)	–	–	–		3.78	1.35	10.56	
Receives the flu vaccine yearly								
No	1.0	-		0.0125	1.0	–		0.0143
Yes	2.30	1.20	4.43		2.28	1.18	4.41	
Wears protective eye mask or face shields								
No	1.0			< 0.0001	1.0	–		< 0.0001
Yes	4.40	2.34	8.24		6.65	3.22	13.71	

#### Factors associated with compliance with wearing protective eye mask or face shields before and after the COVID-19 outbreak

Before the COVID-19 outbreak, the following factors significantly increased compliance of using eye mask or face shields: female vs male (OR = 2.399; 95% CI 1.22–4.72), sterilizing general dental examination instrument with autoclave vs others (OR = 10.81; 95% CI: 1.87–62.72), sterilizing dental extraction instruments with autoclave compared with non-autoclave techniques (OR = 0.11; 95% CI: 0.02–0.68), and use of disinfectant to clean impression materials vs no use or other disposal methods (OR = 2.09; 95% CI: 1.16–3.75). Dentists who worry about getting AIDS during dental treatment were 1.76-fold more likely to wear eye and face shields compared with those did not worry (OR = 1.75; 95% CI: 1.01–3.04). Those wearing hair caps were 4.34-fold (OR = 4.34; 95% CI: 2.19–8.59) more likely to wear protective eye mask or face shields compared with those who do not wear hair caps. After the COVID-19 outbreak, females (OR = 4.20, 95% CI: 1.61–10.96), those who use disinfectant to clean impression materials (OR = 2.84, 95% CI: 1.45–5.59), and those who wear hair caps (OR = 5.38, 95%CI: 2.72–10.62) remained significance factors associated with the use of protective eye mask or face shields. Additionally, those dentists who never treated AIDS patients were more likely to wear protective eye mask or face shields (OR = 2.61; 95% CI: 1.24–5.47) compared with dentists who had treated AIDS patients (Table 4).

**Table 4:** Factors associated with compliance with wearing protective eye mask or face shields before and after the COVID-19 outbreak

Variables	Before COVID-19 Wear protective eye mask or face shields				After COVID-19 Wear protective eye mask or face shields			
	Adjusted odds ratio		95% CI	P-value	Adjusted odds ratio		95% CI	P-value
Gender								
Male	1.0			0.0113	1.0			0.0065
Female	2.40	1.22	4.72		4.20	1.61	10.96	
General dental examination instruments								
Others	1.0			0.0079	–	–	–	–
Autoclave	10.81	1.87	62.72		–	–	–	–
Extraction instruments								
Others	1.0			0.0180	–	–	–	–
Autoclave	0.11	0.02	0.68		–	–	–	–
Disinfection of impression materials								
No or other disposal method	1.0			0.0137	1.0			0.0037
Use of disinfectant	2.09	1.16	3.75		2.84	1.45	5.59	
Have treated AIDS patients								
Yes	–	–	–	–	1.0	1.22		0.0078
Never	–	–	–	–	2.61	1.22	5.47	
Do not know	–	–	–	–	0.91	0.45	1.85	
Worry about getting AIDS during dental treatment								
Do not worry	1.0			0.0475	–	–	–	–
Worry	1.75	1.01	3.04		–	–	–	–
Wearing hair caps								
No	1.0			< 0.0001	1.0			< 0.0001
Yes	4.34	2.19	8.59		5.38	2.723	10.618	

#### Factors associated with compliance with surface disinfection of working tables before and after the COVID-19 outbreak

Before the COVID-19 outbreak, factors, such as bur disinfection (OR = 2.33; 95% CI: 1.25–4.32), the use of a biological indicator during sterilization of implanted devices (OR = 1.94; 95% CI: 1.08–3.50), and keeping treatment tables clear (OR = 2.13; 95% CI: 1.42–3.19), were associated with compliance regarding disinfection of the surface of working tables. After the COVID-19 outbreak, use disinfectant to clean impression materials (OR = 3.22, 95% CI: 1.01–9.70) and keeping treatment tables clear were associated with compliance regarding surface disinfection of working tables (Table 5). The disinfection of working table surfaces was associated with compliance regarding keeping treatment tables clear before and after COVID-19, and undergoing regular flu vaccination (OR = 6.11, 95% CI: 2.89–12.92) was identified as an additional factor after the COVID-19 outbreak (Table 6).

**Table 5:** Factors associated with compliance with surface disinfection of working tables before and after the COVID-19 outbreak

Variables	Before COVID-19 Surface disinfection of working tables				After COVID-19 Surface disinfection of working tables			
	Adjusted odds ratio		95% CI	P-value	Adjusted odds ratio		95% CI	P-value
Bur disinfection								
Others	1.0			0.0076	–	–	–	–
Autoclave	2.33	1.25	4.32		–	–	–	–
Biologic indicator use during sterilization of implanted devices								
No	1.0			0.0276	–	–	–	–
Yes	1.94	1.08	3.50		–	–	–	–
Disinfection of impression materials								
No or other disposal method	–	–	–	–	1.0			0.0041
Use of disinfectant	–	–	–	–	3.22	1.07	9.70	
Keep treatment tables clear								
No	1.0			0.0003	1.0			< 0.0001
Yes	2.13	1.42	3.19		4.37	2.31	8.27

**Table 6:** Factors associated with compliance with keeping treatment tables clear before and after the COVID-19 outbreak

Variables	Before COVID-19 Keeping treatment tables clear				After COVID-19 Keeping treatment tables clear			
	Adjusted odds ratio		95% CI	P-value	Adjusted odds ratio		95% CI	P-value
Receives the flu vaccine yearly								
No	–	–	–	–	1.0			< 0.0001
Yes	–	–	–	–	6.11	2.89	12.92	
Disinfection of working table surfaces								
No	1.0			< 0.0001	1.0			0.0459
Yes	2.86	1.69	4.84		1.95	1.01	3.74	

## Discussion

The current study investigated the impact of COVID-19 on the knowledge, attitude and behaviors of infection control among dentists. The study also identified factors associated with compliance regarding behaviors to control the COVID-19 pandemic. Comparison of the responses to the surveys regarding infection control practices between 2018 and 2020, we found that knowledge of HIV infection and the window of HIV transmission was significantly increased in the pre-COVID-19 period compared with post-COVID-19 period. The use of PPE, hair caps, and protective eye mask or face shields was also greatly improved during the COVID-19 pandemic. The same phenomenon was also found regarding the practice of table surface disinfection and keeping the treatment table clear when not in use. The factors associated with the use of hair caps and protective eye mask or face shields differed between the pre- and post-COVID-19 periods.

Our study found a high level of knowledge on viral infection control (HIV/AIDS transmission) given that greater than 80% of dentists answered correctly in both time periods. The result is consistent with previous studies performed in different countries [23, 24]. HIV has been recognized worldwide since its emerged in 1980, and several countries have implemented safety standards and developed regulatory organizations to ensure infection control practice and education, thus increasing awareness and knowledge of the importance of the disease control [25]. One explanation of the high viral infection control knowledge in Taiwan is that Taiwan experienced the SARS epidemic in 2003. Education of infection control pertaining to the etiology of SARS was introduced, thereby increasing knowledge [20].

We further demonstrated that knowledge of infection control was higher in the post-COVID 19 period compared with the pre-COVID-19 pandemic period. The rapid of distribution of news and reports from Taiwan’s mass media on SARS may have contributed to fears and attitudes about SARS control measures [20]. We hypothesize that the global COVID-19 pandemic, which was widely reported on all sources of media around the world, could prompt people and health care workers to seek information on viral infection and standard preventive control measures. Our hypothesis is favored by our result regarding the increased number of Taiwanese dentists reporting correct responses regarding information regarding COVID-19 infection (94.76%) and concern (94%) of being infected with COVID-19. This finding is consistent with numerous studies from different nations that have also reported good COVID-19 knowledge among dentists, including those in Lebanon (91.3%), Pakistan (93.2%) and various continents (92.7) [26–28]. In fact, previous studies claimed that greater than 70% of dentists used official government websites as the main source of information on COVID-19. This finding imply that dentists are now seeking COVID-19 knowledge [26]. However, the rate of correct answers regarding the HIV/AIDS transmission window and COVID-19 disinfection protocols are quite unsatisfactory. This finding suggests ample room for improving knowledge.

The WHO has recommended the use of PPE, including surgical masks, non-surgical masks, gloves, goggles, face shields, gowns and N95 masks, in the context of COVID-19. In addition to standard PPE, hair caps are also used by dentists [26, 29]. A study in Lebanon reported that 77.7% of dentists wore dental goggle, mask, gloves, face shields, head covers and feet covers [26]. This finding is consistent with our study, which revealed that greater than 95% of dentists in Taiwan wore gloves, mask and protective uniforms. This finding is in contrast to that reported in Iran, where a low percentage of dentists wore PPE (44%). The low rate of PPE usage in Iran was due to an increased demand for masks, which caused increased prices and ultimately a shortage of masks [30]. However, this situation was not observed in Taiwan [10].

Interestingly, we found significant results regarding the use of caps and protective eye masks or face shields between pre-and post-COVID-19 period. According to previous reports, COVID-19 is transmitted through droplets and direct or indirect contact. In addition, the transmission route is not restricted to the respiratory tract, and the virus can enter the human body through the eyes [31]. Therefore, the characteristics of COVID-19 transmission increased the numbers of dentists using hair caps, protective eye mask or face shields during the COVID-19 pandemic.

We also found that a greater number of dentists comply with environmental disinfection protocols (disinfection of working table surfaces and keeping treatment tables clear when not in use). This finding is not surprising given that studies have revealed that the virus that causes COVID-19 can persist for up to 2–10 days on inanimate surfaces, thus facilitating its spread via droplets and contaminated hands or surfaces [32, 33]. We further investigated factors that were associated with wearing hair caps and eye mask/face shield. We observed that better compliance with wearing hair caps before the COVID-19 outbreak is significantly related to annual flu vaccination and the use of protective eye mask or face shields. After the COVID-19 outbreak, hospital setting, use of chemical indicators, half-cut compartments, and COVID-19 related information sources are factors that significantly affect the use of hair caps.

During the SARS-CoV-2 outbreak, many of studies reported hospital-acquired COVID-19 infection [34, 35], and most COVID-19 information was reported by television outlets. In addition, previous studies have shown that dentists have a better compliance rate for infection control in larger dental institutions (such as teaching institutions or hospitals). This finding may be explained by the fact that as the experience of the dental worker increases, the cumulative number of trainings related to infection control guidance also increases, which is likely to improve infection control compliance [36, 37]. Thus, we found that individuals who worked in the hospital and did not receive information from the Dental Association were more likely to wear hair caps. In addition, those who use chemical indicators and half-cut compartments potentially considered themselves more vulnerable to COVID-19 infection and therefore used hair caps.

Our findings also show that the following factors are significantly related better compliance with wearing protective eye mask or face shields in the post-COVID-19 period: females, disinfection of impression materials, never treated AIDS patients, and use of hair caps and waterproof isolation gowns. Dentists are involved in many aerosol-generating procedures that represent one possible mode of transmission [38]. Thus, dentist who wear hair caps and waterproof isolation gowns are more likely to wear of a face shield and protective eye mask.

In addition, our research results also show that better compliance with wearing a protective eye mask or face shield is significantly related to the following factors: female, disinfection of impression materials, and have not treated AIDS patients. Females exhibit better compliance for wearing basic protective barriers compared with males [39]. Askarian et al. observed that dentists in Iran exhibit moderate to extremely high fears and anxiety of transmitting HIV to oneself or other patients and rejecting HIV/AIDS patients [24, 40]. We believe that those dentists who have never treated AIDS patients in our study are aware and scared of being infected with HIV. Thus, these dentists consequently used protective eye mask or face shields.

Disinfection of impression materials is a control practice that is commonly used together with the use of hand hygiene protocols as well as PPE and protective clothes among staff members [26]. This finding may imply that those who are willing to follow control measures also use PEE. This finding could support our result that disinfection of impression materials is associated with the use of eye protection and face shields.

Finally, Taiwan Centers of Disease Control issued the “Guidance for Dental Response to COVID-19 Infection Control Measures” in August 2020 to strengthen dentists’ compliance with infection control [41]. The Science and Technology Research Project entitled “Establishing a self-assessment mechanism for dental infection control operations” was implemented in 2018. In addition, a dental self-assessment policy was added, and re-education training and on-site visits to dental medical institutions were held to enhance dentists’ awareness and implementation of infection control. Thus, in the context of Taiwan dental care, procedures have been implemented to maintain and ensure a proper response to the outbreak of infectious diseases.

Our study has some possible limitations. First, approximately 40% of dentists failed to return the questionnaire, which may lead to a nonresponse bias. We used a nationally representative sample of dentists to investigate changes in compliance with the recommended infection control measures to minimize deviation. In addition, although there is no statistically significant differences between the original regional sample and the regional sample returned for the 2020 survey based on the analysis of consistency according to the goodness of fit test. However, a statistically significant difference is noted between the regional distribution of the questionnaires returned in 2018 and the original sampling design (P = 0.02). The proportion of the questionnaire returned from the Central District is greater than the proportion of the original questionnaire. In contrast, the proportions of 2020 survey respondents from the Southern and Kaohsiung Pingtung districts are lower than that noted for the original survey, which may cause bias.

Second, research results may be affected by response bias given that the survey results are based on self-reported data. Third, recall bias may also occur given the retrospective nature of investigation. We investigated the events that occurred during the previous 12 months and reviewed the experience of the dental practice many years ago. Fourth, the COVID-19 pandemic was less serious in Taiwan compared with other countries. During the survey response period, no large-scale community infection cases were reported in Taiwan. Thus, these results should not be generalized to other countries. Finally, the surveys were separated by 2 years. During this two-year period, the government and dental organizations have successively introduced policies and continuing education courses to improve infection control. These new policies and courses may also affect the results 2 years later; thus, these results are not entirely due to the changes caused by the COVID-19.

## Conclusion

Based-on our nationwide survey among dentists in Taiwan, we found a positive impact of COVID-19 on knowledge, attitude and behavior regarding to infection control. Although compliance with recommended infection control measures has been significantly improved over time, compliance with the use of hair caps, protective eye mask or face shields and environmental disinfection as well as knowledge regarding infectious diseases still need to be improved. In addition, although our study revealed that the COVID-19 epidemic has had a certain degree of influence on recommended infection control measures, the exact influencing factors still require further research. A qualitative study is needed to explain in detail how and why dentists comply with these infection control behaviors. Dentist face a high risk of infection by a contagious disease. Thus, dentists must maintain good infection control practices.

## Supplementary Information

**Additional file 1.** **Figure S1:** Number of confirmed cases in Taiwan during the COVID-19 pandemic in 2020.

## Data Availability

The datasets used and/or analyzed during the current study are available from the corresponding author on reasonable request.
