# Supplementary material for: The impact of COVID-19 on knowledge, attitude, and infection control behaviors among dentists
Source: BMC Oral Health. 2021 Nov 19;21:584. doi: 10.1186/s12903-021-01946-w (PMC8602991; doi:10.1186/s12903-021-01946-w)
Supplement: Supplementary file 1 — Additional file 1. Figure S1: Number of confirmed cases in Taiwan during the COVID-19 pandemic in 2020. [file 12903_2021_1946_MOESM1_ESM.docx]

Additional Figure S1: Number of confirmed cases in Taiwan during the COVID-19 pandemic in 2020
